# Bioactive Peptides from Walnut Residue Protein

**DOI:** 10.3390/molecules25061285

**Published:** 2020-03-12

**Authors:** Xiangyang Li, Manli Guo, Jingtian Chi, Jiangang Ma

**Affiliations:** 1 Science and Technology Department, Hebei Lvlei Agroforestry Technology Co., Ltd. Shijiazhuang 050050, China; 2 College of Food Science and Engineering, Shandong Agricultural University, Taian 271018, China

**Keywords:** walnut residue, bioactive peptide, antioxidation, antihypertensive activity, preparation

## Abstract

Walnut residue is a kind of high-quality plant protein resource. The bioactive peptide prepared from walnut residue has excellent health care functions such as antioxidation and antihypertensive activity, but at present, walnut residue is often regarded as waste or low value feed, fertilizer and other materials. The uneconomical use of walnut residue has hindered the development of the walnut industry to some extent. Effective utilization of walnut residue protein to develop bioactive peptides and other products is of great significance to realize the comprehensive utilization of walnut residue, improve the added value of by-products, and change the current low utilization rate of walnut residue. In this paper, the preparation, purification and structure identification of walnut protein bioactive peptides are reviewed, and different functional walnut active peptides (WBPs) are introduced. The potential effects of these bioactivities on human health and their different uses in food, medicine and other industries are discussed. The purpose is to provide reference information for the effective utilization of walnut residue resources and the development of walnut industry.

## 1. Introduction

Walnut (*Juglans mandshurica*) is the most widely distributed nut in the world, and also considered the oldest nut in the world, originating in East Asia, Southeast Europe and North America [1,2]. Walnuts are rich in unsaturated fatty acids, proteins, polyphenols and minerals [3,4] with antioxidant [5], antitumor [6], anti-inflammatory [7], cholesterol reduction [8,9], blood pressure reduction and cardiovascular disease risk reduction [10] and other important biological activities. The lipid content of walnut kernel is as high as 52–74% [11], which can be processed into walnut oil. Walnut oil is rich in polyunsaturated fatty acids, which is popular with consumers, and its demand is increasing year by year. The increase of walnut oil market demand leads to the increase of walnut residue, a by-product of walnut oil.Walnut residue contains about 40–45% protein [12]. Walnut protein is mainly composed of albumin, globulin, gliadin and glutenin. There are 18 amino acids in walnut protein, and the content of eight kinds of essential amino acids meets the needs of the human body [13,14], which is close to the standard recommend by the World Health Organization (WHO), Food and Agriculture Organization (FAO) and United Nations University (NUN) in 2007 [15] and it is considered to be an important plant protein source for human food and a plant protein with great development potential (Table 1).

Aranz et al. reported that walnut residue contributed the most to walnut antioxidant activity, and walnut oil contributed less than 5% of walnut total antioxidant activity [17], which provided enough information for walnut residue active ingredient extraction. However, walnut residue is often regarded as waste or low value feedstuff, fertilizer and other materials. The uneconomical use of walnut residue has hindered the development of walnut industry to some extent.

Bioactive peptides are composed of 2 to 20 amino acids linked by peptide bonds, andare of particularly concern in the field of nutrition and food science. It is one of the most popular research topics and functional factors with great development prospects in the international food industry [18]. Antioxidant peptides, antihypertensive peptides, immunoactive peptides and neuroactive peptides are some of the most common bioactive peptides. The main source of bioactive peptides is food protein from animal or plant, which is obtained by enzymolysis, fermentation, or in vivo or in vitro digestion. In recent years, the research on the development and deep processing of walnut residue protein is deeper and deeper. Researchers have used different technologies to prepare bioactive peptides from walnut residue (protein). Walnut bioactive peptides (WBPs) with different physiological functions have been continuously prepared. As a new type of walnut residue deep processing product, walnut bioactive peptide (WBP) has broad application prospects and market potential. This review summarizes the current WBP preparation, separation, purification and structure identification technology, discusses the functionality of these bioactive peptides, their potential impact on health and their different uses in food and medicine industries, in order to provide reference information for the efficient use of walnut residue resources and the development of walnut industry.

## 2. Preparation of WBP

There are many methods for the preparation of bioactive peptides from plants, such as enzymatic methods, fermentation methods and chemical methods. Chemical methods involve hydrolyzing protein to produce active peptides by acid or alkali catalysis. The reaction process is difficult to control, and the environmental friendliness is low, so it is not suitable for the industrialization of WBP. At present, the bioactive peptides from walnut meal are mainly prepared by enzymatic and fermentation methods.

### 2.1. Enzymatic Preparation

Due to the advantages of protease, such as specificity, high efficiency, mild reaction conditions, easy control, no nutritional loss and so on, the enzymatic method is widely usedfor the preparation of WBP. The enzymes used for the preparation of WBP must follow the same requirements as other industrial enzymes, which requires not only a wide range of sources, but also a relatively low price. According to the literature, the commonly used walnut proteolytic enzymes are pepsin [19,20,21], trypsin [22,23], alkaline protease [24], papain [25], neutral protease [26], composite protease (pancreatin) [21], etc. As special proteins with physiological activity, enzymes have different activation centers and hydrolysis specificity. Different proteases can be selected to hydrolyze the same protein, and their hydrolysis effects are different. Even if the same enzyme is selected to hydrolyze the same protein, the hydrolysis effect will be different under different environmental conditions and different degrees of enzymolysis. Therefore, the control of enzyme conditions, such as enzyme species selection, pH, temperature, time, ratio of material to liquid, enzyme activity and ratio of enzyme to substrate, is very important for the preparation of WBP.

The natural walnut protein has a compact stereostructure, and most of its cleavage sites are contained in the protein, which makes it difficult to be hydrolyzed by protease. In order to solve this problem, Lv et al. [27] and Li et al. [28] studied the effect of ultrasonic assistance on the enzymatic hydrolysis of walnut residue. The results showed that ultrasonic-assisted hydrolysis of walnut protein had a positive effect on the reaction characteristics and energy efficiency. The degree of hydrolysis (DH) of WBP by ultrasonic assisted enzymatic hydrolysis was significantly improved. compared with conventional enzymatic hydrolysis, the DH of WBP by ultrasonic-assisted enzymatic hydrolysis and ultrasonic pretreatment followed by enzymatic hydrolysis increased by 1.43 and 0.71 times, respectively. Not only that, but also the biological activity of the hydrolysate was improved, which can significantly improve the growth and cell activity of yeast, promote the accumulation of glycogen and trehalose, and improve the taste of beer [28]. As far as we know, high-pressure homogenization, microwave and other pretreatment assisted enzymatic hydrolysis technologies have been successfully applied to the preparation of plant-derived active peptides such as soybean [29], peanut [30], but rarely used in the preparation of active peptides from walnut meal protein pretreatment.

In addition, some bioactive peptides prepared by enzymatic methods often have strong ability to chelate metals such as calcium and zinc [31,32]. Liao et al. reported that WBP-Zn, a walnut protein zinc-chelating peptide prepared by combining the WBP obtained by an enzymatic method with zinc ion has a better ability of reducing toxicity and better anti-proliferation effects [32]. Yang et al. found that the WBP prepared by an enzyme method has strong iron chelating ability, and the smaller the molecular weight, the stronger the iron chelating ability. The WBP iron chelating peptide prepared by chelating WBP with ferrous ion has excellent antioxidant and antibacterial activities, which shows better absorption effect than the conventional iron supplement in model gastrointestinal digestion experiments. They also found that WBP not only has strong ability to chelate iron, but also has a certain ability to chelate calcium, and has potential to develop new organic calcium supplements [33]. At present, a large number of people suffer from zinc, calcium and iron deficiency, so it is very important to develop excellent metal supplements. The chelates of WBPs and metal ions have more excellent functions [32,33]. Obviously, the application of WBPs prepared by enzymatic method in the preparation of peptide metal chelates provide a great possibility for the development of excellent metal supplements.

### 2.2. Fermentation Preparation

The production of bioactive peptides by fermentation combines the production of protease with the enzymatic hydrolysis of protein raw materials. This can not only reduce the production cost of bioactive peptides, but also produce certain detoxification and debittering effects on protein raw materials in the fermentation process, which lays a foundation for effectively promoting the efficient utilization of protein resources and the safe production of bioactive peptides. Therefore, the production of WBP by fermentation has been widely studied in recent years. At present, there are two kinds of fermentation methods: solid fermentation and liquid fermentation. 

**Figure 1:**
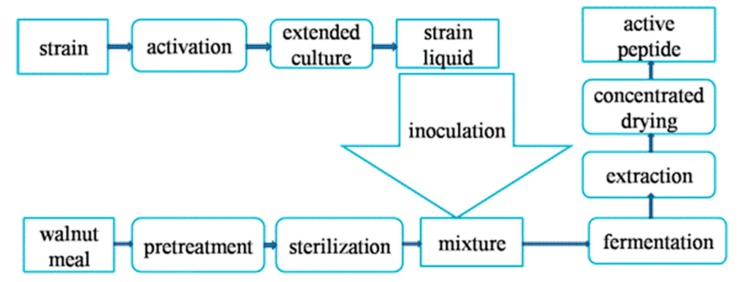
Technological process of preparing WBP by solid fermentation.

Gao et al. optimized the technological conditions for the preparation of WBP by liquid fermentation of walnut residue with *Bacillus natto*. The specific fermentation process after optimization is as follows: the cold pressed walnut residue is crushed through a 40 mesh screen, and then the walnut powder, NaCl and glucose are weighed in, respectively, according to amass ratio of 200:1:1. The mass concentration of the substrate is adjusted to 80 g/L by adding water, the pH value is adjusted to 8.0 by NaOH, and then a high-pressure sterilization pot is used for wet and hot sterilization at 121 °C for 15 min. After cooling, the walnut powder solution is inoculated with *Bacillus natto* seed solution according to a 11% mass ratio, and fermented in a 33 °C constant temperature oscillator at 150 r/min. WBP is separated and purified after 84 h fermentation. Under the optimized conditions, the mass concentration of WBP was 2.58 mg/mL, and the degree of hydrolysis reached 37.5%. [34]. Wu et al. optimized the technological conditions for solid-state fermentation of walnut protein residue by *Bacillus subtilis*, under which the DH of walnut protein could reach 41.8%. The specific process steps are as follows: walnut residue, KH_3_PO_4_ and MgSO_4_ are mixed according to a mass ratio of 13:2:1 to prepare walnut residue solid culture medium with water content of 1.50 mL/g. The solid culture medium is sterilized at natural pH and 121 °C for 20 min. Then, the suspension of *Bacillus subtilis* is put into the solid-state fermentation medium according to the inoculation amount of 10.50% of the mass ratio, and fermented at 37 °C for 82 h. At the end of fermentation, the enzyme in solid-state fermentation medium is inactivated at 121 °C for 10 min. After centrifugation and microfiltration, a walnut peptide solution could be obtained [35]. Zheng, Li and Ding successfully prepared an ACE inhibitory peptide by solid-state fermentation of defatted walnut residue with *Bacillus subtilis* [36]. It should be pointed out that the solid fermentation system has certain advantages over the liquid fermentation system, specifically in the higher enzyme productivity [37], more stable fermentation process [38], higher environmental friendliness [39], which is more suitable for extracting walnut residue resource active peptide. Figure 1 shows the technological process of WBP preparation by solid fermentation.

## 3. Isolation and Purification of WBP

The hydrolysate of walnut protein with biological activity obtained by enzyme or fermentation is composed of active peptides with different molecular weight, macromolecular protein, insoluble substrate and soluble impurities. In practical research and production, it is often necessary to obtain active peptides with higher levels of an active component and purer active peptide by separation and purification technology suitable for the target product. As far as we know, membrane separation technology (ultrafiltration) and chromatography technology are widely used in the separation and purification of WBPs.

Generally, the first step of isolation and purification of WBP is ultrafiltration. The hydrolysate of walnut protein passes through the ultrafiltration membrane with a certain pore size under pressure driving, and WBP can pass through the ultrafiltration membrane smoothly because of its small particle size, while insoluble substrate, enzyme and protein with larger molecular weight remain outside the membrane to achieve the purpose of preliminary purification of walnut peptide. The preliminary purified walnut peptide can pass through the ultrafiltration membrane with different pore sizes in turn, and can also complete the separation of active peptides with different molecular weight ranges [12,19,36,40]. However, with ultrafiltration technology is difficult to achieve the effective separation of peptides with small or very similar molecular weights. In practical research, it is often combined with chromatography technology.

Usually, the second step of isolation and purification of WBPs is chromatography. The more commonly used chromatography methods in the separation and purification of WBPs are gel permeation chromatography (GPC) [40], ionexchange chromatography (IEC) [40], high performance liquid chromatography (HPLC) [41], reverse-phase high-performance liquid chromatography (RP-HPLC) and metal affinity chromatography (MAC) [26].

It should be pointed out that the above mentioned separation and purification technologies of WBP have their own advantages and disadvantages. A single separation and purification technology cannot fully meet the requirements of high-efficiency separation and purification of WBP. In the practical work of separation and purification of WBP, so researchers often consider a variety of properties of WBP comprehensively, and adopt some multi-method combination technology to separate and purify it, so as to achieve better separation and purification effect [20,40,41].

## 4. Identification of Walnut Active Peptide

In order to further explore the functional mechanism of WBP, analyze the qualitative relationship between the structure and function of WBP, and develop walnut peptide products with stronger physiological activity, the purified walnut peptide often needs further structural analysis and identification. The classical method for structural identification of walnut peptide is to use Edman sequencing technology to identify the amino acids in the walnut peptide from N-terminal in turn, and get the amino acid sequence of peptide chain [41]. This method is time-consuming and insensitive. Mass spectrometry and other hyphenated techniques can complement Edman sequencing. At present, they are widely used in the analysis of amino acid sequence of walnut peptide, such as electrospray ionization mass spectrometry (ESI-MS) [24,36], matrix-assisted laser desorption/ionization time-of-flight mass spectrometry (MALDI-TOF-MS)[40,41], quadrupole time-of-flight mass spectrometry (Q-TOF-MS) [21,42], determination of clenbuterol by liquid chromatography-tandem mass spectrometry (LC-MS/MS) [19] and so on. In recent years, de novo sequencing method has shown significant advantages in the structural identification of walnut peptides [24,36,43,44]. This technology can complete the de novo sequencing of unknown walnut peptide without any protein database, with high accuracy and sensitivity.

## 5. WBPs with Different Functions

### 5.1. Antioxidant Peptide

Since Harman put forward the theory of free radicals, it has been recognized that the free radicals produced by oxidation in human body are related to human aging and many diseases [45]. The research on reactive oxygen species and free radicals has also become afrontier and hot spot of modern life science. Therefore, the preparation and extraction of antioxidants have developed rapidly, and the use of antioxidants has become more and more extensive. They are not only used as antioxidants in food processing, but also as functional factors in the development of health food and cosmetics. There are many kinds of antioxidants. However, the chemical synthesis of antioxidants such as BHA, BHT, etc. has its own toxic and side effects that are not conductive to human health. Governments of various countries have issued mandatory policies to regulate their acceptable daily intakes (ADI) value, and excessive addition is strictly prohibited. Based on the above reasons, screening and evaluation of natural resources with strong antioxidant activity has become a new trend in biological, medical and food science research. Antioxidant peptide is a kind of bioactive peptide with antioxidant function, which can effectively remove the excess active oxygen free radicals in the body, protect the normal structure and function of cells and mitochondria, prevent the occurrence of lipid peroxidation, and then play the role of delaying aging and preventing diseases [45,46]. In addition, it also has many advantages, such as high water solubility, low foaming, high safety, wide application range, etc. Therefore, it has a good market prospect to develop and utilize high-efficiency antioxidant peptides.

In recent years, walnut residue, as a potential plant protein resource, has attracted more and more attention. Many researchers have successfully extracted walnut peptide products with strong antioxidant activity from walnut protein, characterized the structure of these products, identified and isolated a number of small peptides with antioxidant activity (Table 2), and explored and discussed the antioxidant mechanism of walnut peptide.

The mechanism of antioxidant action of bioactive peptides, the relationship between antioxidant function and the molecular structure of bioactive peptides has been explored more and more. It is reported that the antioxidant properties of active peptides are closely related to many factors, such as their ability to remove reactive oxygen, the ability to donate hydrogen atoms or transfer electrons to free radical compounds, and the ability to chelate transition metals such as copper or iron [45,49]. Antioxidant peptides can scavenge free radicals by inhibiting hydrogen atom transfer and electron transfer, or prevent the production of free radicals and active oxygen based on instantaneous metal ion chelation [50,51]. The scavenging ability of active peptides to 2,2′-azino-bis (3-ethylbenzthiazoline-6-sulfonicacid) cation radicals (ABTS^+^), hydroxyl radicals (.OH), 1,1-diphenyl-2-picryl-hydrazyl free radicals (DPPH), and other radicals, the scavenging ability of active peptides to superoxide and other active oxygen and the chelating ability of active peptides to metal ionsare the scientific evaluation criteria for the antioxidant ability of active peptides. In addition, the amino acid composition, sequence, molecular size and other information of the active peptide are very important to explore the molecular mechanism of the antioxidant activity of the active peptide. Gu et al. isolated fourteen new antioxidant peptides from walnut protein trypsin hydrolysate, identified and analyzed the structure of these peptides, found that most of these fourteen antioxidant peptides contain tyrosine or cysteine, and speculated that tyrosine and cysteine may contribute a lot to the antioxidant activity of the active peptide. The possible mechanism is that cysteine residue has reductive SH group and potential antioxidant activity, while tyrosine has the ability of donating hydrogen atom, which is a good material to remove ABTS^+^ and other free radicals [12,52]. Feng et al. isolated six walnut peptides from the simulated digestion products of walnut meal protein, and characterized their antioxidant activity and molecular structure, respectively. According to the structure-activity relationship of the six peptides, they proposed that the amino acid sequence and composition of the peptides play an important role in the antioxidant activity [21]. The report from Chen et al. [43] is slightly different from that of Feng et al. [21]. Chen et al. identified seventy-seven peptides from the trypsin hydrolysate of defatted walnut residue, and isolated WSREEQEREE and ADIYTEEAGR, two walnut peptides with strong protective effect on PC12 cells damaged by H_2_O _2_. They found that the amino acid mixture with the same composition as these two peptides had no protective effect on H_2_O_2_ damaged cells, so they proposed that the main factor affecting the antioxidant effect is the amino acid sequence, not the amino acid composition [43]. Sheng et al. identified more than 1000 peptides from walnut hydrolysates by de novo sequencing. On this basis, they established a scoring method based on structure and activity, and screened eight new antioxidant peptides of walnut. It was found that the content of Glu, Arg, ASP, Gly and other specific amino acids was closely related to the scavenging ability of hydroxyl radicals of peptide [44]. As far as we know, the antioxidant mechanism of walnut peptide has not been completely cleared up at present, and further research is needed.

In addition, many literatures reported that walnut peptides with high antioxidant activity also have excellent anti-tumor activity [22,24], improve memory activity [43,48,53,54], enhance immunity and anti-fatigue activity [54], etc. Wang et al. found that the mixed solution of low molecular weight walnut peptide improved the memory deficit behavior induced by sleep deprivation in rats (666 mg/kg peptide daily recommended). They identified three highly active neuroprotective peptides (GGW, VYY and LLPF) from the solution. Their experimental results showed that these three peptides could effectively protect PC12 cells from apoptosis induced by glutamine, and the relative survival rate of cells were 78.29 ± 3.09%, 80.65 ± 1.74% and 83 97 ± 3.06%. In addition, all of these peptides can reduce the consumption of antioxidant enzymes (SOD and GSH PX), inhibit the production of ROS, regulate the expression of apoptosis related proteins (Bax and Bcl-2) [48]. Therefore, walnut peptide may be considered as a potential health care product against neurodegenerative diseases related to memory impairment. Ma et al. found that the walnut peptide prepared by papain enzymatic hydrolysis of walnut protein under the optimum conditions (substrate concentration 5%, enzyme substrate ratio 10%, temperature 60 °C, time 3 h) not only had superior antioxidant activity, but also showed excellent anti-cancer activity. The amino acid sequence of walnut peptide with high anticancer activity purified by them is CTLEW, with a molecular weight of 651.2795 Da. It is a new type of biological peptide with amphiphilic structure, which can induce apoptosis and autophagy of cancer cells, significantly inhibit the growth of cancer cells, but has no cytotoxic effect on normal cells. In addition, CTLEW can promote the proliferation of mouse spleen lymphocytes and the secretion of IL-2, enhance the phagocytic ability of mouse macrophages, and improve the immunity of the body [47]. Fang et al. reported that low molecular weight (is less than 3 kDa) WBPS (800 mg/kg daily recommended) can significantly extend the swimming time of mice, increase the liver glycogen content in mice, reduce the lactic acid content in mice, increase the immune organ index of mice, promote the proliferation of mouse intestinal T lymphocytes, and has significant anti-fatigue and immunomodulatory effects [46]. These results show that walnut protein extracted from walnut residue can be hydrolyzed moderately to prepare highly active anticancer peptide, and walnut protein hydrolysates and purified anticancer peptides can be fully considered for the development of functional foods and anticancer drugs.

### 5.2. Antihypertensive Active Peptide

Hypertension is a global public health problem [55]. Angiotension-converting enzyme (ACE) is a kind of dipeptidyl carboxypeptidase, which plays a role in the renin-angiotension system and the kallikrein-kinin system, leading to the rise of blood pressure.ACE inhibitory peptide can inhibit the activity of ACE, reduce the tension of blood vessels, blood volume and blood pressure, and play an important physiological role in regulating human blood pressure. The ACE inhibitory peptide extracted from food protein can effectively reduce high blood pressure, but it has no effect on normal blood pressure. More importantly, the ACE inhibitory peptide extracted from food has no side effects compared with antihypertensive drugs [56]. More and more attention has been paid to the safety of ACE inhibitory peptide and its beneficial effect on human health. Since Oshima and others first reported the successful extraction of food-derived ACE inhibitory peptide in 1979 [57], a large number of food-derived ACE inhibitory peptide products have been found. 

In recent years, ACE inhibitory peptide prepared from walnut residue has been widely concerned because of its low cost, convenient separation and purification, excellent antihypertensive effect, and effective utilization of waste resources. Wang etal. reported that walnut protein hydrolysate can significantly reduce the systolic blood pressure of hypertensive mice (*P* < 0.05), with strong ACE inhibitory activity and stability [20]. Jahanbani et al.reported that with the increase of the concentration of walnut protein trypsin hydrolysate, the *K_m_* of ACE increased gradually, while *V_max_* decreased gradually, which indicated that the presence of walnut protein hydrolysate (ACE inhibitory peptide) reduced the affinity of ACE with substrate, thus inhibiting its activity and playing a role in reducing blood pressure [58]. Wang et al. compared the ACE inhibitory activity of LPGRPPIKPWP obtained from walnut protein hydrolysate before and after simulated gastrointestinal digestion in vitro. It was found that the 50% inhibitory concentration (IC50) values of LPGRPPIKPWP before and after digestion were 0.136 and 0.173 μM/mL, respectively, which confirmed that the peptide was relatively stable during digestion and had the potential of ACE inhibitory activity in vivo [20]. Zheng, Li and Ding reported that the component with molecular weight less than 1kDa in the walnut residue extract fermented by *Bacillus subtilis* had the largest ACE inhibitory activity [36]. In a word, more and more ACE inhibitory peptides from walnut residue have been successfully prepared, separated and purified (Table 3). These ACE inhibitory peptides have been fully considered for application in functional food and drug processing.

At present, the focus of most of the research on ACE inhibitory peptides has gradually changed to the structure-activity relationship of peptides, the inhibition mechanism of peptides on ACE and the industrialization of ACE inhibitory peptides, and more and more in-depth. A large number of literatures have reported that the chain length, amino acid composition, amino acid sequence and spatial structure have a great influence on the activity of ACE inhibitory peptide.

Long peptides are difficult to bind to the active sites of ACE, so most of the high active ACE inhibitory peptides have a chain length of 2-12 amino acids, the lower the molecular weight (<3kDa), the higher the inhibitory activity [36,60,61]. However, in many cases, the influence of amino acid type and sequence on ACE inhibitory activity of peptide is much greater than that of peptide chain length. Different amino acid types of ACE inhibitory peptide have different inhibitory activities. The amino acid types of ACE inhibitory peptide are the same, but the sequence is different, and their inhibitory activities will be significantly different [59]. Generally proline, branched amino acids and aromatic amino acids may contribute to the ACE inhibitory activity of peptides [62]. Wang et al. used the Lineweaver-Burk plot analysis method to explore the inhibition mode of walnut short peptide EPNGLLLPQY on ACE, and found that EPNGLLLPQY showed a mixed inhibition mode on ACE. Specifically, EPNGLLLPQY can not only combine with ACE at the active site to prevent ACE lyses, but also combine with ACE at the inactive site and prevent the interaction of ACE with the substrate through spatial bit resistance effect, so as to achieve effective suppression of ACE activity. In addition, Wang et al. further analyzed the interaction mechanism between the docking receptor ACE and the ligand EPNGLLLPQY by means of molecular docking, and found that the ACE inhibitory peptide was a linear peptide, which further proved that the spatial structure and molecular weight of the peptide played an important role in the ACE inhibitory activity of the peptide, and found that there were three strong interactions between EPNGLLLPQY and ACE. It is hydrogen bond, hydrophobic and electrostatic interaction. Sixteen hydrogen bonds, seven hydrophobic interactions and three electrostatic interactions were found at the interaction sites. These strong interactions between EPNGLLLPQY and ACE contributed the most to the ACE inhibitory activity of walnut peptide [23].

### 5.3. Xanthine Oxidase Inhibitory Peptide

Nowadays, the incidence of hyperuricemia is increasing. Hyperuricemia is closely related to hypertension, atherosclerosis, insulin resistance and kidney disease, which seriously affects health [63,64,65,66]. Drugs usually used to treat hyperuricemia have some side effects that may affect the health of the body. Therefore, the development of natural and efficient xanthine oxidase inhibitors (XOI) is very important for the health of hyperuricemia patients [67]. In recent years, the excellent XOI activity of some food-derived bioactive peptides has been widely studied. Li et al. reported that walnut meal hydrolysate and dephenolized walnut meal hydrolysate (DWMH) have superior XOI activity, which can effectively reduce the serum uric acid level in hyperuricemia rats. Two new anti hyperuricemic peptides, WEPKDEX and ADIYTE, with molecular weights of 640.8 Da and 710.7 Da respectively, were successfully isolated and purified from DWMH, showing very high XOI activity, with IC_50_ values of 17.75 mg/mL and 19.01 mg/mL, respectively [68]. Based on the above work, Li et al. further studied the XOI activity and mechanism of walnut active peptide in vitro. It was found that walnut active peptide containing Trp can effectively inhibit xanthine oxidase, and the increase of Trp quantity will significantly improve the XOI activity of Trp containing peptide. Similar to allopurinol, Trp has a positive interaction with the key residues of xanthine oxidase, such as Glu802, Leu873, etc., so as to effectively inhibit the activity of xanthine oxidase [69]. Su et al. disclosed a method for preparing bioactive peptides with the effect of reducing uric acid from walnut residue. The target bioactive peptides obtained by this method can significantly reduce the level of serum creatinine in rats, and have a certain degree of protection on the renal function of rats, with significant effect of reducing uric acid [70]. It is clear that walnut XOI active peptide has the potential to introduce functional food processing to improve the condition of hyperuricemia patients.

## 6. Conclusions and Prospects

Walnut oil, as a high-quality functional oil, is more and more favored by consumers, and its annual consumption is also increasing year by year, so the output of walnut residue, a by-product of processing, is also increasing year by year. Walnut residue is a kind of high-quality plant protein resource, which contributes the most to the antioxidant activity of walnut. However, walnut residue is often regarded as waste or low-value feed, fertilizer and other materials. The uneconomical use of walnut residue hinders the development of walnut industry to some extent. The effective use of walnut residue protein to develop walnut residue protein products is of great significance to realize the comprehensive utilization of walnut residue, improve the added value of by-products and change the current situation of low utilization of walnut residue.

### 6.1. Market Prospect of WBP

Due to the serious aging of the society, there are many elderly people who are threatened by memory impairment, cancer, hypertension, hyperlipidemia, poor digestion and absorption ability and other diseases. In addition, there are many people who are troubled by high blood pressure, hyperlipidemia and other diseases because of malnutrition caused by eating high-fat food, junk food or over processed food. In view of the special needs of these people, it is very necessary to develop food-based health food and functional drugs to provide them with nutrition and health care. Walnut peptide has reasonable amino acid composition, no cholesterol, small molecular weight, easy to absorb, can effectively improve protein energy malnutrition, with excellent antihypertensive, anti-cancer, anti-fatigue, memory improving, immunity improving and other functions; it is especially suitable for the development of health food and medicine for the above special population. Not only that, but now consumers’ awareness of the positive correlation between bioactive peptides and health is constantly improving [71], and the global market demand for bioactive peptides has been growing steadily, so the market prospect of walnut peptide products is broad. According to statistics, the total output of the global walnut market in 2016 was 72.521 million tons. Based on the walnut peptide extraction rate of 25%, 1 million ton of walnut residue can produce more than 25 million tons of WBP products, and it is preliminarily estimated that the economic value of the products after being put into the market will be about 200 billion dollars. The functional foods, health foods, and medicines that are further developed with WBP as raw materials will create greater economic benefits after being widely put on the market.

### 6.2. Research Direction of Walnut Peptide in the Future

The nutritional quality, processing adaptability, storage stability, biological activity and other physical and chemical characteristics of walnut active peptide products are closely related to the preparation process. The selection of protease and fermentation strains, hydrolysis conditions and degree of hydrolysis control, separation and purification technology will affect the composition, structure and function of the final product. In the future, it is necessary to further explore the qualitative and quantitative relationship between the structure, function and preparation process of walnut peptide, innovate the preparation process of walnut peptide, and develop a new preparation process of walnut peptide with higher bioavailability and more significant function.

At present, a large number of WPB products with anti-oxidant activity, anti-hypertensive activity, anti-hyperuricemia activity, memory improvement activity, anti-tumor activity, etc. have been identified and developed, and have been fully considered for widely used in functional food and pharmaceutical processing. However, with the exception of a few industrialized products, most products are still in the laboratory or pilot stage, and there are few WBP products on the market. As far as we know, most of the WBP products reported in the literature use in vitro or animal experiments to evaluate their activity and safety, and the lack of sufficient evidence of human studies for effectiveness and safety is the main obstacle to commercialization of products. Many official organizations such as the European Union, the United States, and China have clear legal requirements that require food-derived biopeptides to have sufficient characteristics in human physiology. Animal and in vitro studies can be used as background information to explain the peptide’s mechanism of action, but cannot be used as valid evidence. Without adequate human safety and efficacy data, manufacturers’ active peptide products will be rejected for marketing applications. In order to realize the commercialization of WBP products as soon as possible, food scientists should pay more attention to the data collection of safety, effectiveness, absorption and metabolism of WBP in clinical trials in the future, and draw reliable conclusions that support the beneficial effects of walnut peptides on humans. So as to realize the commercialization of various WBP products as soon as possible and give full play to their contribution to human health.

## Figures and Tables

**Table 1:** Amino acid composition (g/100 g) of degreased walnut powder and walnut protein [13,15,16].

Amino Acids	DFWF	WPC	WPI	WHO/FAO/UNU (2007) *			
				1–2	3–10	11–14	Adults
Met	1.16 ± 0.02 ^b^	0.99 ± 0.08 ^b^	1.44 ± 0.10 ^a^				1.6
Val	4.18 ± 0.21 ^b^	3.50 ± 0.18 ^c^	4.62 ± 0.10 ^a^	4.2	4.0	4.0	3.9
Thr	3.58 ± 0.21 ^a^	2.55 ± 0.09 ^b^	3.30 ± 0.12 ^a^	2.7	2.5	2.5	2.3
His	2.38 ± 0.13 ^a^	1.89 ± 0.09 ^b^	2.30 ± 0.10 ^a^	1.8	1.6	1.6	1.5
Ile	3.28 ± 0.10 ^b^	3.03 ± 0.09 ^b^	3.99 ± 0.06 ^a^	3.1	3.1	30	3.0
Leu	7.13 ± 0.07 ^a^	5.64 ± 0.12 ^b^	7.29 ± 0.08 ^a^	6.3	6.1	6.0	5.9
Lys	2.58 ± 0.05 ^a^	2.01 ± 0.07 ^b^	2.13 ± 0.11 ^b^	5.2	4.8	4.8	4.5
Cys	0.84 ± 0.06 ^a^	0.70 ± 0.12 ^b^	0.81 ± 0.15 ^a^				0.6
Phe	4.94 ± 0.05 ^a^	3.49 ± 0.07 ^b^	4.61 ± 0.12 ^a^				3.8 **
Tyr	2.76 ± 0.22 ^b^	2.30 ± 0.13 ^b^	3.21 ± 0.09 ^a^				3.8 **
Trp	5.50 ± 0.02			7.4	6.6	6.5	6.0
Pro	4.22 ± 0.07 ^a^	2.33 ±0.04 ^c^	3.18 ± 0.01 ^b^				
Ala	4.74 ± 0.05 ^a^	3.31 ± 0.15 ^b^	4.29 ± 0.11 ^a^				
Arg	14.73 ± 0.09 ^a^	11.24 ± 0.18 ^b^	14.81 ± 0.11 ^a^				
Gly	5.43 ± 0.15 ^a^	3.54 ± 0.11 ^c^	4.18 ± 0.08 ^b^				
Ser	5.84 ± 0.19 ^a^	3.96 ± 0.22 ^c^	5.15 ± 0.07 ^b^				
Glu	22.16 ± 0.12 ^a^	15.78 ± 0.20 ^c^	19.49 ± 0.11 ^b^				
Asp	10.04 ± 0.21 ^a^	6.95 ± 0.19 ^c^	9.38 ± 0.11 ^b^				

DFWF, defatted walnut flour; WPC, walnut protein concentrate, WPI, walnut protein isolate; Met, methionine; Val, valine; Thr, threonine; His, histidine; Phe, phenylalanine; Ile, isoleucine; Leu, leucine; Lys, lysine; Pro, proline; Cys, cysteine; Tyr, tyrosine; Trp, tryptophan; Ala, alanine; Arg, argnine; Gly, glycine; Ser, serine; Glu, glutamic acid; Asp, aspartic acid; * Values in these columnsrepresentthe WHO/FAO/UNU (2007) recommended patterns of essential amino acids for 1–2, 3–10, 11–14 year oldchildren and adults. ** The total amount of Phe and Tyr is 3.8g/100g. ^a,b,c^ Numerical values with different letters in the same row are significantly different(*P*-value < 0.05).

**Table 2:** Some walnut antioxidant peptides that have been identified.

Hydrolase	SIT	ACS *	RMM orM/C	Ref
Pepsin	RP-HPLC-ESI-MS	ADAF	423.23 Da	[19]
Trypsin	UPLC-ESI-MS / MS	WSREEQEREE	1377 Da	[43]
Trypsin	UPLC-ESI-MS / MS	ADIYTEEAGR	1124 Da	[43]
Neutral protease	RP-HPLC,nanoLC-ESI–MS/MS,de novo sequencing	LAGNPDDEFRPQ	1357.6262 Da	[26]
Neutral Protease	RP-HPLC,nanoLC-ESI–MS/MS, de novo sequencing	VEDELVAVV	971.5080 Da	[26]
Papain	RP-HPLC	CTLEW	651.2795 Da	[47]
Tripsin, Viscozyme L	UPLC-ESI-Q-TOF-MS/MS	GGW	319.1400 Da	[48]
Tripsin, Viscozyme L	UPLC-ESI-Q-TOF-MS/MS	VYY	444.2129 Da	[48]
Tripsin, Viscozyme L	UPLC-ESI-Q-TOF-MS/MS	LLPF	489.3071Da	[48]
Pancreatin	ESI-MS/MS	YS	269.11 *m*/*z*	[12]
Pancreatin	ESI-MS/MS	YSVH	505.23 *m*/*z*	[12]
Pancreatin	ESI-MS/MS	YK	310.14 *m*/*z*	[12]
Pancreatin	ESI-MS/MS	YT	283.13 *m*/*z*	[12]
Pancreatin	ESI-MS/MS	LPC	331.16 *m*/*z*	[12]
Pancreatin	ESI-MS/MS	EM	279.13 *m*/*z*	[12]
Pancreatin	ESI-MS/MS	CA	192.14 *m*/*z*	[12]
Pancreatin	ESI-MS/MS	SQK	362.3272 *m*/*z*	[12]
Pancreatin	ESI-MS/MS	CR	288.2900 *m*/*z*	[12]
Pancreatin	ESI-MS/MS	CHC	362.3265 *m*/*z*	[12]
Pancreatin	ESI-MS/MS	GHC	316.3200 *m*/*z*	[12]
Pancreatin	ESI-MS/MS	YA	253.1181 *m*/*z*	[12]
Pancreatin	ESI-MS/MS	YG	239.1025 *m*/*z*	[12]
Pancreatin	ESI-MS/MS	NW	318.3007 *m*/*z*	[12]
Alcalase and protamex	HPLC-FTMS, de novo sequencing, scoring method	VEGNLQVLRPR		[44]
Alcalase and protamex	HPLC-FTMS, de novo sequencing, scoring method	LAGNPHQQQQ		[44]
Alcalase and protamex	HPLC-FTMS, de novo sequencing, scoring method	HNLDTQTESDV		[44]
Alcalase and protamex	HPLC-FTMS, de novo sequencing, scoring method	AGNDGFEYVTLK		[44]
Alcalase protamex	HPLC-FTMS, de novo sequencing, scoring method	WSVWEQELEDR		[44]
Alcalase and protamex	HPLC-FTMS, de novo sequencing, scoring method	QQRQQQGL		[44]
Alcalase and protamex	HPLC-FTMS, de novo sequencing, scoring method	AELQVVDHLGQTV		[44]
Alcalase and protamex	HPLC-FTMS, de novo sequencing, scoring method	EQEEEESTGRMK		[44]
Alcalase and protamex	HPLC-FTMS, de novo sequencing, scoring method	VEDELVAVV		[44]
Alcalase	LC-ESI-QTOF	AGGA		[42]
Simulating gastrointestinal digestion	RP-HPLC-UPLC-QTOF-MS	TY		[21]
Simulating gastrointestinal digestion	RP-HPLC-UPLC-QTOF-MS	SGGY		[21]

The SIT, ACS, RMM, M/C andRefin the table refer to separation and identification technology, amino acid sequence, relative molecular mass, mass/charge ratio and reference respectively. * The amino acid sequence is in the form of 20 single letter abbreviations, namely G—Gly, A—Ala, V —Val, L—Leu, I—Ile, P—Pro, S—Ser, T—Thr, H—His, E—Glu, D—Asp, Q—Gln, N—Asn, K— Lys, R—Arg, C—Cys, M—Met, F—Phe, Y—Tyr and W—Trp.

**Table 3:** ACE inhibitor peptides that have been identified.

Pre-Met	SIT	ACS *	RMM	IC_50_	Ref
Enzymolysis	UF, GPC-HPLC, MALDI-TOF-MS	TWPERPPQIP	1033.42Da	25.67 μg/mL	[40]
Enzymolysis	UF, GPC, HPLC (RP-HPLC), ESI	YEP	407.43Da	0.29 μmol/L	[24]
Enzymolysis	MALDI-TOF-MS, GPC-HPLC	LPGRPPIKPWPL	1353.67 Da	128.98 μg/mL	[41]
Enzymolysis	RP-HPLC, HPLC	YVPHWDL	929Da	0.136–0.173 μm/mL	[20]
Fermentation	UF, UPLC-ESI-MS/MS, RP-HPLC	VQTL LGYEN	459.35Da 594.28Da	- -	[36]
Enzymolysis	UPLC-Q-TOF-MS/MS, HPLC-MS/MS	EPNGLLLPQY	1142.60 Da	0.233 μm/mL	[23]
Enzymolysis	UPLC-MALDI-TOF-MS	LY	295.19Da	0.042 μm/mL	[59]
Enzymolysis	UPLC-MALDI-TOF-MS	YLA	366.24Da	0.396 μm/mL	[59]

The Pre-met, SIT, ACS, RMM and Ref in the table refer to preparation method, separation and identification technology, amino acid sequence, relative molecular mass and reference, respectively. * The amino acid sequence is in the form of 20 single letter abbreviations, namely Gly—G, Ala—A, Val—V, Leu—L, Ile—I, Pro—P, Ser—S, Thr—T, His—H, Glu—E, Asp—D, Gln—Q, Asn—N, Lys—K, Arg—R, Cys—C, Met—M, Phe—F, Tyr—Y and Trp—W.
